# Drugs for the Quorum Sensing Inhibition of Oral Biofilm: New Frontiers and Insights in the Treatment of Periodontitis

**DOI:** 10.3390/pharmaceutics14122740

**Published:** 2022-12-07

**Authors:** Alessandro Polizzi, Martina Donzella, Giada Nicolosi, Simona Santonocito, Paolo Pesce, Gaetano Isola

**Affiliations:** 1 Department of General Surgery and Surgical-Medical Specialties, School of Dentistry, University of Catania, Via Sofia 78, 95125 Catania, Italy; 2 Department of Surgical Sciences (DISC), University of Genova, 16132 Genoa, Italy

**Keywords:** periodontitis, quorum sensing, quorum quenching, periodontal therapy, quorum sensing inhibitors

## Abstract

Chemical molecules are used by microorganisms to communicate with each other. Quorum sensing is the mechanism through which microorganisms regulate their population density and activity with chemical signaling. The inhibition of quorum sensing, called quorum quenching, may disrupt oral biofilm formation, which is the main etiological factor of oral diseases, including periodontitis. Periodontitis is a chronic inflammatory disorder of infectious etiology involving the hard and soft periodontal tissues and which is related to various systemic disorders, including cardiovascular diseases, diabetes and obesity. The employment of adjuvant therapies to traditional scaling and root planing is currently being studied to further reduce the impact of periodontitis. In this sense, using antibiotics and antiseptics involves non-negligible risks, such as antibiotic resistance phenomena and hinders the re-establishment of eubiosis. Different quorum sensing signal molecules have been identified in periodontal pathogenic oral bacteria. In this regard, quorum sensing inhibitors are emerging as some interesting solutions for the management of periodontitis. Therefore, the aim of this review is to summarize the current state of knowledge on the mechanisms of quorum sensing signal molecules produced by oral biofilm and to analyze the potential of quorum sensing inhibitors for the management of periodontitis.

## 1. Introduction

Periodontal disease consists of chronic inflammation of the tooth-supporting tissue caused by specific oral microorganisms leading to periodontal destruction and alveolar bone loss [1]. The World Health Organization (WHO) estimated that 5–20% of the worldwide adult population is affected by severe periodontitis (probing depth [PD] ≥6 mm), 35–50% by mild periodontal disease [2] and it is a major cause of tooth loss in adults [3]. Therefore, periodontitis as a diffuse disease among nations is an important health and economic challenge in modern society [4].

Evidence supports that periodontitis may induce a systemic inflammatory state that contributes to an increased risk of developing various diseases, among which cardiovascular and metabolic diseases [5,6], but also obesity, autoimmune disorders (including rheumatoid arthritis), Alzheimer’s disease and some types of cancers [7]. More specifically, recent research tried to identify specific inflammatory pathways of periodontal disease that may be involved in the increasing risk and/or worsening of other systemic diseases [8].

In this regard, increasing evidence is showing that periodontitis etiopathogenesis is complex and multifactorial. The primary cause is the accumulation of dysbiotic biofilm on oral surfaces due to poor oral hygiene [9]. Research in the periodontal field was initially aimed at identifying bacterial strains with greater aggressiveness and virulence, capable of promoting the transition from simple gingivitis, related to the accumulation of bacterial plaque, to a subgingival inflammatory state that involves all periodontal tissues [10]. The so-called “*Red-complex bacteria*” (*Porphyromonas gingivalis*, *Prevotella intermedia*, *Tannerella forsythia*, and *Treponema denticola*) have been recognized as the major pathogenic microorganisms implicated in periodontal disease pathogenesis [11,12]. It has been recognized that oral bacterial species are capable of organizing themselves into complex communities known as biofilms [10].

Subsequently, research focused on investigating the role of the host’s immuno-inflammatory response and it has been revealed that predisposed individuals, in the presence of pathogenic periodontal biofilm, respond more aggressively, leading to the more severe and rapid destruction of periodontal tissues [13,14]. Therefore, periodontal disease results from an abnormal host immuno-inflammatory response and dysbiotic microorganisms organized in biofilms. These bacteria are able to damage periodontal tissues through the production of enzymes and metabolites and, consequently, fibroblasts and leukocytes release different proinflammatory mediators such as metalloproteinases (MMPs), prostaglandins, proteolytic enzymes, reactive oxidative species (ROS) and cytokines [15,16,17,18]. In particular, gingival keratinocytes, fibroblasts and macrophages’ proinflammatory activities are stimulated by interleukin (IL)-1, IL-6, and IL-17 alone or in synergy with IL-1β, tumor necrosis factor (TNF)-α, toll-like receptors, and prostaglandin E2 (PGE2) [19]. The trigger of this local inflammation results in periodontal attachment and alveolar bone loss [20].

For these reasons, periodontitis is now considered a disease with multifactorial etiology and the result of genetic, microbial and environmental factors. Therefore, the progression and severity of the disease is the result of both the pathogenicity of the oral biofilm, the host’s immune-inflammatory response and the interaction of various environmental factors such as smoking and/or the presence of systemic diseases [21].

New understandings are emerging about how oral bacterial species organized in biofilms are able to communicate with each other. In this regard, quorum sensing (QS) has been defined as the process by which microorganisms monitor and regulate their population density through chemical signaling [22]. This chemical signaling system allows the bacteria to coordinate their behavior and to increase their survival skills through the growth and synthesis of the matrix biofilm, greatly improving their resistance to conventional therapies such as antibiotics and antiseptics [23].

Non-surgical periodontal therapy (scaling and root planing—SRP) is the elective treatment for periodontal disease. However, mechanical instrumentation can induce a significant reduction of pathogenic periodontal bacteria; the long-term effects are limited [19]. Furthermore, periodontal pockets with probing depths ≥6 mm are more refractory to healing with conventional therapy alone, therefore, various therapeutic approaches have been proposed, such as surgical therapy, the use of systemic antibiotics and adjunctive therapies [24,25,26]. However, a recent Cochrane review reported that there is very low-certainty evidence (for long-term follow-up) that adjunctive systemic antibiotics are of any help for non-surgical periodontal treatment [27]. The unresolved periodontal inflammation may favor the presence of residual pockets and periodontal pathogenic bacteria capable of triggering the processes of destruction of periodontal tissues [28].

In this regard, new therapeutic approaches are being studied that are effective and able to overcome systemic antibiotics’ limitations and side effects to increase the benefits of SRP. Among these, the potentialities of using QS inhibitors (which act with mechanisms called quorum quenching—QQ) have been emerging recently [23,29].

Therefore, the aim of this review is to summarize the current state of knowledge on the mechanisms of quorum sensing signal molecules produced by oral biofilm and to analyze the potential of quorum sensing inhibitors for the management of periodontitis.

## 2. Materials and Methods

A detailed literature review of the current knowledge of periodontitis and QS in the oral cavity was performed, using the databases MEDLINE (through PubMed) and Web of Science.

## 3. Quorum Sensing Signaling in Microbial Biofilm

**Figure 1:**
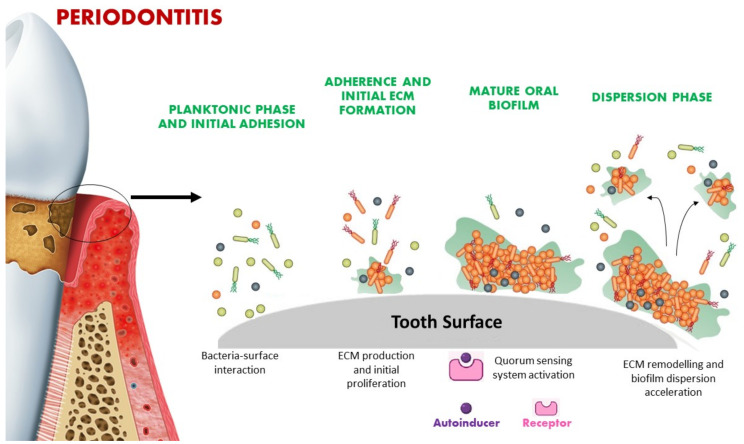
QS chemical signaling varies in relation to population density. The binding of AIs with bacterial receptors can favor the organization of planktonic bacteria into complex and organized structures known as biofilms that underlie some oral disorders such as periodontal disease. ECM: extracellular matrix.

Oral bacteria live and develop in organized structures called “biofilms”, which consist of specialized microbial communities attached to oral surfaces and immersed in a self-produced extracellular matrix [30]. Biofilm’s protective structure makes bacterial communities more resistant to the action and penetration of external physical and chemical agents such as antibiotics and oral antiseptics. In this regard, biofilm formation is regulated by chemical signaling. More specifically, through the release of small signaling molecules, oral bacteria can communicate with each other—the process called QS—to regulate gene expression in terms of population density, acid resistance, virulence and biofilm development [29,30,31]. The signaling molecules, called autoinducers (AIs), favor the establishment of a real social community able to coordinate its growth and development through the density-dependent stimulation of cell metabolism and the formation of a new protective matrix [23] (Figure 1). When the stimulatory threshold is reached, oral bacteria encounter a change in gene expression [32].

Oral bacteria involved in the QS system mainly show three capabilities: (1) secretion of chemical signaling molecules, (2) detection of the alterations in concentrations of these molecules, and (3) response to these signals through the regulation of gene transcription [33]. The QS system and AIs secretion depend on the bacterial cell density. At a low cell density and planktonic phase, AIs are not much expressed and just diffuse away. However, at high cell density (this could, for example, be favored by poor oral hygiene) AIs concentration may exceed the threshold level and determine an alteration of gene transcription [34] (Figure 1).

The first example of QS was observed in the second half of the 1960s, by studying the luminescent bacterium *Vibrio fischeri*, which is able to emit light only when its concentration exceeds a certain threshold.

This mechanism allows the bacterium to become luminescent only when they are present in large numbers, preventing wasting energy when the population is too small to emit a visible signal. Nealson et al. [35] discovered from cultures of this bacterium that the chemical signal, necessary for the production of luminescence derived from a substance, designated as autoinducer-1 (AI-1), which was an acyl homoserine lactone (AHL). It was found that the sensory mechanism produced in response to this signal basically consists of the activity of two proteins, LuxR and LuxI. LuxR is a transcriptional regulator that, following the binding with the autoinducer, determines the expression of the luciferase operon; LuxI catalyzes a new synthesis of the acyl-homoserine lactone [35].

Subsequently, the second group of acyl-homoserine lactone-dependent QS systems was identified in another marine Vibrio. Two proteins are involved in this system, LuxM and LuxN, which are partly similar to LuxI and LuxR. In fact, LuxM synthetizes acyl homoserine lactone; instead, LuxN is a sensor kinase that binds to the acyl-homoserine lactone and ultimately activates the LuxO response regulator [36]. It was established that gram-negative bacteria mostly communicate via AHLs-dependent systems. Conversely, gram-positive bacteria predominately communicate with small peptides (AIP) that often contain chemical modifications [37,38]. Specifically, AIP is detected by two-component membrane-bound histidine kinase receptors [39]. Among the two-component signal transduction systems (TCSTSs), several have been reported in the oral cariogenic *Streptococcus mutans* (*S. mutans*). One, encoded by a system called “comABCDE”, was found to regulate the excretion of a signal molecule, competence-stimulating peptide (CSP), depending on specific cell density levels and environmental stresses [40,41,42]. In addition to being involved in QS mechanisms, CSP also induces genetic competence in *S. mutans.* In this regard, Li et al. [41] proved that genetic transformability in cells derived from biofilms was 10- to 600-fold greater than for planktonic cells. Moreover, this system is also important for the initiation of biofilm formation, the expression of bacteriocins, and the regulation of acid tolerance in *S. mutans* [43,44,45]. Finally, other QS intraspecies pathways for communication between *S. mutans* cells are the XIP-ComRS signaling system and the PdrA/WGK system [46,47]. AHLs and small peptides-dependent systems are generally considered species-specific.

In 1994, Bassler et al. found that *Vibrio harveyi* controlled the expression of luminescence through a different quorum sensing system encoded by the luxPQ genes and responded to another autoinducer, designed as AI-2 [48]. AI-2 are a group of signaling molecules derived from a furanosyl borate diester, the product of the LuxS enzyme. They are common to both gram-positive and gram-negative bacteria, for inter-species exchanges [32].

Many authors examined the production of QS systems signaling molecules by oral microorganisms, involved in both periodontitis and dental caries. Bacteria most frequently present in sites with active periodontitis have been studied in order to know how their QS mechanisms are able to influence numerous bacterial and host activities [49].

Frias et al. showed that three genera of periodontal isolates, *Fusobacterium nucleatum* (*F. nucleatum*), *Prevotella intermedia* (*P. intermedia*), and *Porphyromonas gingivalis* (*P. gingivalis*), had an AI-2 activity [50]. *P. gingivalis* and another periodontal pathogen, *Treponema denticola* (*T. denticola*), can interfere with quorum-sensing-dependent virulence properties in *S. mutans* [51]. Other studies showed that also *Aggregatibacter actinomycetemcomitans (A.a)* produces AI-2 as its signaling molecules. For example, it was demonstrated that the release of the AI-2 from *A. actinomycetemcomitans* leads to a decrease in biofilm formation by *C. albicans* and an increase in the biofilm formation by *S. mutans* [52].

The same conclusion also concerns some species of *Streptococci*, such as *S. mutans* and *S. gordonii* [53]. For example, *S. mutans* can cross-communicate with *C. albicans* [54].

Regarding AHLs-dependent QS, data suggest that these signaling molecules may not be common between bacteria of dental biofilm and periodontal pathogens, despite their presence being discovered on saliva samples [55,56]. Table 1 summarizes the QS systems.

## 4. Quorum Sensing in the Transition from Eubiotic to Dysbiotic Oral Biofilm

Generally, the oral biofilm is mainly composed of commensal species that are harmless towards the host, but it often happens that local or systemic causes can alter this balance, causing a condition characterized by increased proliferation of pathogenic species [58]. The events that most commonly cause changes in the oral microbial community are poor oral hygiene, diet and alterations in the host’s immune defenses.

**Figure 2:**
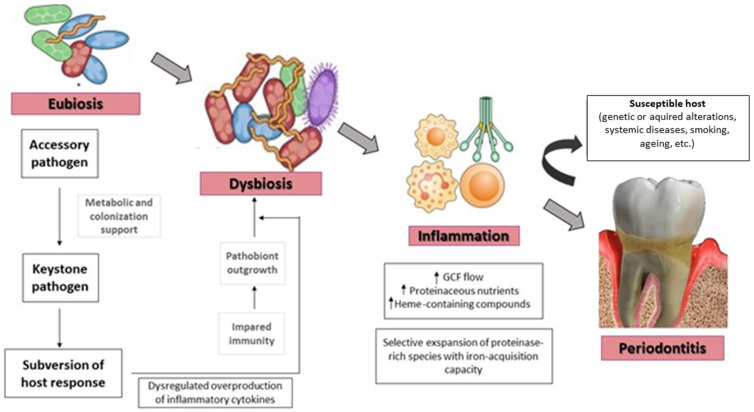
The transition from eubiosis to dysbiosis is favored by several factors. The proliferation of pathogenic species and the impairment of the host response are the starting point. Dysbiosis can then promote inflammation which in susceptible patients can generate periodontitis. In turn, the inflammation can create the conditions necessary for the dysbiosis to continue. GCF: gingival crevicular fluid.

In the dysbiotic state, the microbial community is made up of microorganisms with specific functions and with a high virulence factor, which will allow them to resist for long periods of time [48,49]. Colonization by periodontal pathogens leads to impaired host immune response with a dysregulated overproduction of cytokines and the beginning of the inflammatory process, causing the emergence of dysbiosis. The new environmental conditions generated by the inflammatory process further favor dysbiosis. In fact, inflammation causes an increase in gingival crevicular fluid (GCF), proteinaceous nutrients heme-containing compounds that are used selectively by proteinase rich species associated with dysbiosis. In this way, dysbiosis and inflammation reinforce each other and become the reason why periodontitis becomes a chronic state (Figure 2).

Certainly, quorum sensing mechanisms, facilitating biofilms’ formation, help bacteria perpetuate this dysbiotic state.

**Figure 3:**
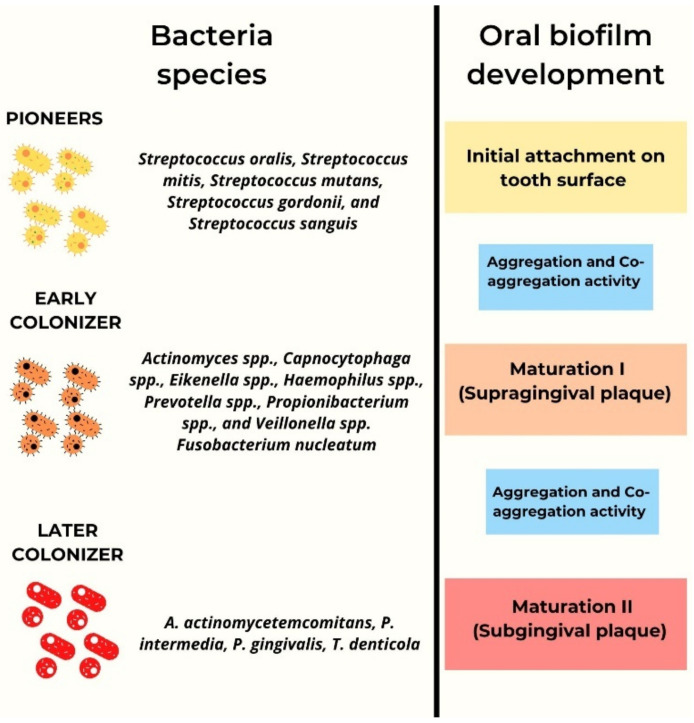
The protagonists of every different phase of oral biofilm development. These are: ‘Pioneers’ which are responsible for the attachment on the surfaces of the tooth, in the first phase; ‘Early colonizers’ which initiate the maturation of the supragingival biofilm, in the second phase; ‘Later colonizers’ which cause the formation of subgingival plaque, in the last phase.

Oral biofilm development consists of phases with different bacterial species as protagonists (Figure 3). In the initial phase, all those bacteria responsible for attachment on the supragingival tooth surface are involved. Among these species, called pioneers, the majority is made up of *Streptococci* spp. (including *Streptococcus oralis*, *Streptococcus mitis*, *S. mutans*, *Streptococcus gordonii*, and *Streptococcus sanguis*). Pioneers together with a number of their substances act as receptors for other bacterial species, called early colonizers, which take over a second phase. This group of bacteria begins to form the biofilm through aggregation and co-aggregation mechanisms [59]. At a later stage, other bacteria, called later colonizers, build a dense network of links with the previous group, especially thanks to the presence of a particular bacterial species, *Fusobacterium nucleatum*. Later colonizers include *Aggregatibacter actinomycetemcomitans* (*A.a.*), *P. intermedia*, *P. gingivalis*, *T. denticola*, and others [60]. Many of them constitute the subgingival plaque, typically composed of gram-negative anaerobic bacteria, which under certain conditions can play a role in the pathogenesis of periodontitis.

Actually, among the various QS systems, the evidence attributes an important role to the AI-2 system. The synthesis of this autoinducer has been identified in both commensal and pathogenic bacteria but in different concentrations. Higher production of pathogens causes a decrease in commensal growth, therefore, a prevalence of pathogens [50,61]. As we said previously, periodontal bacteria have in common AI-2 activity as QS systems, but in each species this auto-inducer can assume a different role.

In *Aggregatibacter actinomycetemcomitans (A.a.)* the presence of AI-2 influences the growth capacity in a biofilm, the virulence and also the aggregation with other species [62]. Regarding biofilm formation and the virulence, several studies have shown that in *A.a.*, as well as in other bacteria, there are at least two different types of periplasmic receptors for AI-2 encoded by lsrB and rbsB. When one or both receptors are inactivated, as a consequence, the growth of the biofilm is reduced or eliminated. These receptors show different kinetics of interaction that allows the bacterium to respond differently depending on the concentration of the autoinducer-2 [63,64]. For this reason, some authors believe that this microorganism can grow in the subgingival biofilm with high cell density and, instead, with low cell density on the surfaces of the oral mucosa [65].

About interaction with other bacteria species, it was demonstrated that serotypes b and f of *A.a.* mediate coaggregation with *F. nucleatum*, thanks to a specific receptor that consist of an O-polysaccharide [66]. In a study conducted on cultured dual-species biofilms on artificial saliva it emerged that *A.a* is able to trigger expression of the QS regulon of *S. mutans* [67].

In addition, AI-2-mediated quorum sensing has been shown to be intimately linked to iron acquisition on both *A.a.* and *P. gingivalis*. Iron is an essential molecule for many bacterial functions, including metabolism and enzyme activity. Iron uptake by *A.a.* depends on the biofilm’s bacterial cell density and autoinducer-2 concentration. In fact, it seems that at high concentrations of AI-2, the bacteria derive iron from the host cells, while in low concentrations of AI-2 they derive it from iron-scavenging chelators [68]. In addition, for *P. gingivalis*, AI-2 regulates different absorption mechanisms, including membrane receptors, depending on the available hemin content, from which this bacterium generally obtains iron. For this bacterium, iron deficiency conditions result in a reduction of its virulence [69]. Other studies demonstrated that AI-2-dependent signaling in *P. gingivalis* is essential for its growth in towering microcolonies; it was found that in co-culture with *S. gordonii*, LuxS and AI-2 of *S. gordonii* are essential for the ability of *P. gingivalis* to produce biofilms [70]. At last, QS mechanisms are positively involved in *P. gingivalis* response to host-induced stresses such as high-temperature exposure, resistance to hydrogen peroxide, and changes in the pH [71].

Autoinducer-2 is also produced by *F. nucleatum, Prevotella intermedia*, and *S. oralis*, but specifically, its role in the biofilm growth of these bacteria has yet to be investigated [50].

Currently, it has only been shown that the AI-2 of *Fusobacterium* can promote its aggregation with the Red complex bacteria, increasing the formation of subgingival biofilm, and that through an interaction with *S. gordoni* and *S. mutans* can also favor their biofilm formation [72,73].

Another periodontal pathogen in which the presence of AI-2 has been identified is *Eikenella corrodens*. In fact, there is evidence that this bacterium expresses a fundamental gene for the production of AI-2 molecules and that the presence of these improves the efficacy of this bacterium in biofilm formation by 1.3 times [74].

Although numerous studies have been performed to demonstrate the production of Acyl homoserine lactone (AHL)-mediated quorum sensing system by oral pathogens [57], even today there is no consistent evidence regarding their presence and their possible role in the development of the oral biofilm [75].

## 5. Antimicrobial Drugs Used for the Prevention and Treatment of Periodontal Disease

Primary prevention of periodontal disease is based on protecting individuals from periodontal pathogens with the aim of preserving their health; but if the pathogen meets the host, it becomes necessary to limit its action and consequently the progression of the pathology, trying to restore a condition of balance and health, even though functional damage may have already occurred. Mechanical control of biofilm can therefore be complemented using specific antimicrobial agents, both preventively and/or therapeutically [76]. These agents can act in a variety of ways: by forming a film along the tooth surface thereby preventing bacterial adhesion, by bactericidal or bacteriostatic effect, by disrupting the chemical bonds between biofilm and surface, by altering the pathogenicity of the biofilm or reinforcing the host’s defensive mechanisms [77].

Antibiotics have long been used and are still a valuable adjuvant to SRP and oral hygiene instruction [78]. The most widely used systemic broad-spectrum antibiotics (e.g., ciprofloxacin, tetracycline, amoxicillin with or without clavulanic acid, azithromycin and doxycycline) act primarily against gram-negatives and obligate anaerobes. Moreover, it has been shown that the same antibiotic molecule is more effective in terms of duration of action with systemic administration compared to local applications.

Systemic antibiotics have greater effects than the topical ones, which have, on the other hand, a limited duration of action. However, side effects and increased bacterial resistance must be taken into account [79]. Metronidazole has a narrower spectrum; it is generally prescribed in combination with amoxicillin and it has been seen that this combination can reduce the need for periodontal surgery and induce long-term stability of the remaining periodontium, by limiting the number of residual pockets after initial therapy [80]. The use of these systemic antibiotics has been associated with less disease progression for 6 months and up to 2 years after a single course and appears to have an effect even at superficial sites [79]. Several studies have shown that systemic antibiotics bring better clinical outcomes when administered immediately after mechanical therapy rather than 3 to 6 months post SRP [81,82] and generally the recommended doses are 250 mg metronidazole and 500 mg amoxicillin, three times daily for 7 days [79,83]. The use of topical antibiotics (e.g., chlorhexidine gluconate, 10% doxycycline hyclate, tetracycline hydrochloride and minocycline hydrochlorid) may be justified by the fewer side effects and would appear to provide additional benefits in reducing probing depth, the plaque score, inflammation and gingival bleeding, compared to SRP alone [84].

Oral antiseptics find wide use in daily oral hygiene practices; they are recommended for all individuals and become necessary for those with disabilities, oral mucositis or other conditions where good oral hygiene needs to be maintained [85]. The most used oral antiseptics include chlorhexidine (CHX), essential oils, triclosan, and quaternary ammonium compounds [86]. CHX is the most studied and believed to be effective oral antiseptic and generally it is formulated as a mouth rinse with a concentration of 0.2% or 0.12%. CHX can bind reversibly to oral tissues and remain there even for more than 12 h. It increases plasma membrane permeability with bacteriostatic effect and alters biofilm formations exerting bactericidal effects at higher concentrations and is also able to lead to the reduction of salivary film formation [87,88]. There are several adverse effects given by the prolonged use of chlorhexidine, among them certainly the most common is tooth staining [89]. Essential oils have excellent anti-inflammatory action based on antioxidant activity and, as well as CHX, result in a reduction in the amount of plaque and appear to improve gingival inflammatory indices [90]. Triclosan is a broad-spectrum antibacterial agent, and as a mouth rinse it seems to exert its action best, leading to significant improvement on plaque and gingival index; it is commercially available with copolymer and sodium fluoride [91]. Quaternary ammonium compounds include cetylpyridinium chloride (CPC), which acts through various mechanisms leading to bacterial cell death, with notable benefits to plaque quantity and inflammatory indicators [92].

The host’s defenses and abilities to respond appropriately to the bacterial attack, that promotes the onset of periodontitis, are critical in promoting or not the progression of the disease, so host modulation has emerged as an additional therapeutic approach [93]. Several recent studies agree that the intake of omega-3 fatty acids for the treatment of periodontitis, either by supplementation or with the diet, can have a positive impact on the disease, with a positive effect on healing in terms of reduction of clinical attachment loss (CAL) and probing depth (PD) [94,95]. Of recent introduction are a class of drugs derived from bacterial species, probiotics (e.g., *Lactobacillus reuteri*), which act on biofilm composition. They appear to be capable of bringing about a reduction in bacterial species and improvements in periodontal tissues; however, it is an evolving topic that needs further study [96,97].

## 6. Quorum Sensing Inhibition Strategies

Due to the increased antibiotic resistance and the difficult removal of the biofilm, several researchers are focusing on new strategies to hinder microorganisms and interrupt this communication, a process referred as ‘quorum quenching’ (QQ) [98]. QQ aims at a preventive action against the formation of biofilm that is more complex to remove once it is stabilized [99]. Because of the multiplicity of pathogenic bacteria that play a role in periodontitis, most QQ research studies related to periodontal disease have been performed on in vitro models, with a limited number of in vivo model studies related mainly to murine species [49].

Various QS inhibition mechanisms have been described; although this is an ever-changing field, the main ones are represented by: the inhibition of signal molecule synthesis in order to block signal generation [100], the enzymatic degradation or the inactivation of signal molecules [100,101], the competitive receptor antagonism to obstruct signal reception [102], the interruption of the signal transduction cascades [103].

The main target in gram-negative bacteria is represented by AHL molecules, which are normally synthesized by a LuxI type enzyme and can diffuse out of the cells [104]. When a high extracellular concentration is reached, the activation of the LuxR receptor protein induces the transcription of specific genes. The AHL system is the basis of multiple pathogenetic behaviors of gram-negative bacteria (e.g., *P. gingivalis*) including host adhesion and biofilm formation, which plays an important role in the onset and progression of periodontal disease [33,105]. Several synthetic analogues of AHLs, obtained by the addition of alcohol or amine groups into the acyl groups, have been tested against *P. gingivalis* biofilms and some of these seem to be particularly effective in reducing the number of biofilm cells, as well as inhibiting the formation of a well-organized biofilm [106].

**Figure 4:**
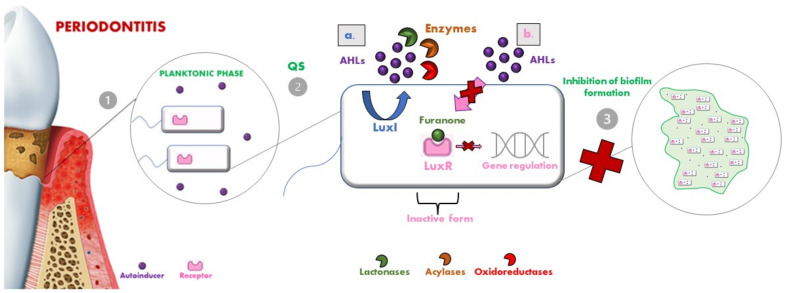
(**1**) Bacteria in the planktonic phase produce autoinducers to communicate with each other. (**2a**) In gram-negative bacteria, AHLs molecules can be degraded by enzymes (lactonases, acylases and oxidoreductases). (**2b**) Halogenated furanones binding to LuxR makes the complex unstable, hindering the binding between signal molecules and receptor, and inhibiting the expression of specific genes. (**3**) This inhibits the formation of biofilm.

The most known mechanism of QQ consists in the enzymatic degradation of AHL molecules. For this purpose are mainly distinguished three groups of enzymes namely lactonases, amidases (or acylases) and oxidoreductases [98] (Figure 4).

These enzymes inactivate signal molecules in different ways:

- Lactonases, by hydrolyzing the ester bond of the lactone ring, cause the consequent degradation of AHLs [107]. Several classes of lactonases have been identified in a large variety, both in bacteria but also in archaea and eukaryotes; some of these prefer AHLs with long acyl chains (e.g., the phosphotriesterase-like lactonases (PLLs) and the paraoxonases (PONs)), while others present a wider spectrum of action (e.g., the metal-β-lactamase-like lactonases (MLLs) and the α/β hydrolase fold lactonases). These lactonases were selected as a model for engineering so that they could be modified to confer enhanced AHL lactonase activity [101];
- Acylases are the other class of QQ enzymes and hydrolyse the amide bond of AHL. In particular, a QQ acylase is represented by PvdQ, which, thanks to its hydrophobic binding pocket, has specificity for long-chain AHLs, and thanks to a targeted engineering approach has changed the range of action also toward shorter AHLs [108,109];
- Oxidoreductases do not degrade the AHL but modify its activity, leading to the inability to bind to the respective receptor [110].

Even natural products (e.g., embeline and piperine) seem to be able to inhibit communication between bacteria (QS), that is, between signal molecules and receptors, hindering the production of biofilm in *S. mutans* [111]. Other plant-derived natural products and phenols have QS-inhibiting abilities, among them coumarin, which can inhibit the formation of *P. gingivalis* biofilms and green tea extracts, which instead would appear to inhibit its growth and adherence [112]. Molecules derived from various plants, algae and fungi (e.g., horseradish–Iberin, garlic–ajoene, sweet basil–osmarinic acid, garlic–disulfides and trisulphides and others) could also inhibit QS, because it has been found that they could act as potential quorum quenchers and be effective against periodontal bacteria and beyond [113].

*Ferula narthex* exudate is rich in umbelliferone derivatives, which would appear to possess antimicrobial activities and moderate antibiofilm potential against clinical strains contained in the dental plaque of diabetic patients with periodontitis [114].

Suppose the signal molecules are reduced in number and the proper concentration is not reached. In that case, the whole process is consequently blocked, so acting in different ways can weaken the structure of an already formed biofilm, such as by furanone [115]. The halogenated furanones are structurally similar to AHLs, binding to LuxR makes the whole complex unstable, thus accelerating the turnover rate of the receptor itself [116,117] (Figure 3). The study of brominated bicyclic synthetic furanones showed a reduction in biofilm biomass and thickness against several periodontal pathogens (e.g., *P. gingivalis*, *F. nucleatum* and *T. forsythia*), without the development of antimicrobial resistance [118,119]. In addition, the combination of brominated furanone and D-ribose in an in vivo murine model showed a reduction in *P. gingivalis* levels and bone loss [120]. A remarkable action, in terms of reduction of *P. gingivalis*, *F. nucleatum* and *T. forsythia* biofilms formation and an inhibition of the interaction of *A. a.* serotypes b and f with *F. nucleatum*, was reported using D-galactose, which thus also showed QQ properties [66,121].

In the form of biofilms, bacterial communities are able to be more resistant and promote the onset of diseases such as periodontal disease [30]. It has been noted that lactonases and acylases can offer excellent help in the prevention of biofilm formation and infection, even for the inserts used for the human body (e.g., vascular prosthesis, urinary catheters) and this result has been reported in *Pseudomonas aeruginosa* and other pathogens [122,123]. Moreover, enzymes are not cytotoxic and have little or no bactericidal properties, making them excellent tools for effective bacterial control [101]. Significant effects were found through the engineering of lactonases, that is, increased activity and stability, both at the level of biofilm formation and significant changes in the structure of the microbial population [124]. One QQ enzyme, the AHL lactonase Aii20j, has proven very effective in inhibiting biofilm formation for in vitro models, both from healthy donors and from patients with oral diseases [23]. Increased bacterial susceptibility to antibiotics has been demonstrated using QS inhibitors and a study has shown that the combined use of antiseptic, antibiotic and QQ compounds appear to yield promising results against *Porphyromonas gingivalis* [106,125].

In gram-positive bacteria, the signal molecules are represented by AIPs. When they reach an appropriate concentration in the environment, they are activated by phosphorylation through proteins with kinase activity, initiating transcription of target genes, therefore, kinase inhibitors interrupt signal transmission. Another mechanism is to block the transcriptional cascade by means of regulators that bind to DNA and inhibit RNAIII production so that different virulence factors cannot be produced [126].

*Streptococcus gordonii*, a commensal gram-positive bacterium, is a pathogen closely related to the onset and progression of several oral diseases, such as periodontitis and dental caries [127,128]. Just as in other streptococci, CSP, a QS molecule, is primarily responsible for biofilm formation [129]. A recent study found that short-chain fatty acids (SCFAs) (e.g., acetate and propionate) effectively inhibited biofilm formation and thus also the occurrence of related oral diseases [130].

The acidogenic bacterial species *S. mutans* is dominant in the etiology of dental caries, so several studies have been conducted to find effective QS inhibition strategies [131,132]. Baicalein, a plant product, showed the ability to interfere in cell communication and the association of CHX with fluoride and trans-cinnamaldehyde resulted in biofilm inhibition of *S. mutans* [133,134]. Several bacterial products have been investigated as anticaries agents (e.g., fructanase, the products of probiotic *Lactobacillus* species) but their role as QS inhibitors needs further investigation [135,136].

An in vitro study investigated the effects of organosulfur compounds and S-Aryl-L-cysteine sulfoxides on biofilm development and QS inhibition on three oral strains (*Streptococcus mutans* UA159, *Streptococcus sanguis* 10556 and *Actinomyces oris* MG1). Six (including L-cysteic acid, diphenyl disulphide, S-(4-tolyl)-L-cysteine) of the analyzed compounds were shown to influence the progression of biofilm maturation and were also able to inhibit *Vibrio harveyi* bioluminescence due to their structural similarity with key molecules in AI-2 pathways [137].

As mentioned above, inhibition of biofilm formation on inserts placed in the human body can offer great help in preventing infection, similarly in clinical dental practice various medical devices are also placed in the oral cavity every day (e.g., dental implants, prostheses, bone grafts, membranes) [138,139]. The abutment surfaces of dental implants are frequently coated by biofilms and the resulting infection can lead to what is known as peri-implantitis, a cause of implant loss or additional surgical maneuvers [140,141]. The use of QS inhibitors could reduce biofilm formation if placed on the abutment surfaces; further studies are needed for this purpose [142]. A recent study found that pre-treatment of titanium discs with D-arabinose for three minutes can inhibit biofilm formation of three species of bacteria, such as *S. oralis*, *F. nucleatum* and *P. gingivalis*, also being an inhibitor of AI-2, it possibly could be used to prevent peri-implantitis and peri-implant mucositis [143]. Table 2 summarizes the QS inhibitors and their mechanism of action.

Effective delivery of these QS inhibitors is important. Various strategies have been thought of and implemented to screen their validity, among them surface coating (e.g., by microparticles containing QQ enzymes) [99,142]; great interest also lies in nanoparticles that would appear to be effective quorum quenchers and are of recent interest, for oral cavity biofilms and beyond [126,144]. However, further research is needed for the entry, delivery and release of the agents to the biofilm matrix [144].

Several of the periodontopathogen bacteria (e.g., *P. gingivalis*, *A.a*) communicate with each other and promote the onset of periodontal infections, even supporting their progression. Routine practices of QS inhibition in combination with oral hygiene procedures (e.g., mechanical plaque removal) could help reduce the severity of periodontal disease or its prevention [113].

There are now several pieces of scientific evidence that, especially in recent years, supports research in favor of QS inhibition strategies; in fact, these enzymes and new drugs could facilitate the targeted treatment of chronic periodontitis. By preventing biofilm formation, better disease control would be achieved [145].

## 7. Conclusions and Future Perspectives

Periodontitis is a multifactorial inflammatory disease in which the bacterial component is necessary but not sufficient for its development. In fact, in the absence of the biofilm with specific pathogenic species, it would not start. The proliferation and diffusion of pathogenic species, together with the alteration of the host response, causes a condition of dysbiosis, which, once established, tends to perpetuate itself over time. It has long been known that bacterial species use a specific language among themselves, with different systems that allow them to communicate and coordinate. Periodontal bacteria also use these quorum sensing mechanisms, especially for oral biofilm development. Since QS activity plays a fundamental role in the pathogenesis of periodontitis, the inhibition of these communication mechanisms can become another important therapeutic strategy. Today, periodontal therapy mainly consists in the mechanical removal of the supra-gingival and sub-gingival biofilm using professional instrumentation and often with adjuvant antibiotic therapy. Currently, research has discovered a number of QS inhibitors, available in nature, produced by plants, fungi, algae, but it is still necessary to carry out numerous studies to demonstrate the real effectiveness of these products in the periodontal area. The biggest challenge is to discover and use molecules and substances that selectively act on pathogenic periodontal bacteria, not interfering with the growth of commensal species. In the future, this type of discovery could partially substitute the use of antibiotics and therefore contribute to reducing the phenomenon of antibiotic resistance.

## Figures and Tables

**Table 1:** Different QS systems in oral bacteria.

System	Molecules	Bacteria Type	Oral Bacteria Species	Mechanism
Acyl homoserine lactone (AHL)	LuxR and LuxI	Gram-negative	Lack of consistent evidence [57]	Species-specific
Acyl homoserine lactone (AHL)	LuxM and LuxN	Gram-negative	Lack of consistent evidence [57]	Species-specific
Small peptides (AIP)	Small peptides	Gram-positive	*S. mutans* [40,41,42]	Species-specific
Furanosyl borate diester (AI-2)	LuxPQ and LuxS	Gram-negative Gram-positive	*F. nucleatum*, *P. intermedia, P.gingivalis*, *T. denticola*, *A.a*, *S. mutans*, *S. gordonii* [50,51,52,53]	Inter-species

**Table 2:** Summary of QS inhibitors in the oral biofilm.

QS Inhibitor	Mechanism of Action	Refs.
Synthetic analogues of AHL	Inhibition of a well-organized biofilm formation against *P.gingivalis*	[106]
Lactonases	Degradation of AHLs (hydrolysis of the ester bond of the lactone ring)	[107]
Acylases	Degradation of AHLs (hydrolysis of the amide bond)	[108]
Oxidoreductases	Inhibition of receptor binding (modification of AHL action)	[110]
Coumarin	Inhibition of the formation of *P.gingivalis* biofilm	[112]
Green tea extracts	Inhibition of *P. gingivalis* biofilm growth and adhesion	[112]
Umbelliferone derivatives	Moderate antibiofilm potential against clinical strains contained in the dental plaque of diabetic patients with periodontitis	[114]
Halogenated furanones	Increased turnover rate of the LuxR receptor (structural resemblance to AHLs)	[116,117]
Brominated bicyclic synthetic furanones	Reduction in biofilm biomass and thickness against *P. gingivalis*, *F. nucleatum* and *T. forsythia*	[118,119]
Brominated furanone and D-ribose	Reduction in *P. gingivalis* levels and bone loss	[120]
D-galactose	Reduction of *P. gingivalis*, *F. nucleatum* and *T. forsythia* biofilms formation and inhibition of the interaction of *A. a.* s serotypes b and f with *F. nucleatum*	[66,121]
Short-chain fatty acids (SCFAs)	Inhibition of biofilm formation of *S. gordonii*	[130]
Baicalein	Inhibition of *S. mutans* biofilm formation	[133]
CHX with fluoride and *trans*-cinnamaldehyde	Inhibition of *S. mutans* biofilm formation	[134]
Organosulfur compounds and *S*-Aryl-L-cysteine sulfoxides	Potential reduction in biofilm formation on *S. mutans* UA159, *S. sanguis* 10556, *Actinomyces oris* MG1 and inhibition of QS on *V. harveyi*	[137]
D-arabinose	Inhibition of biofilm formation of *S. oralis*, *F. nucleatum* and *P. gingivalis* (inhibitor of AI-2)	[143]

## Data Availability

Data are available from corresponding authors upon reasonable request.

## References

[1] Tonetti M.S., Greenwell H., Kornman K.S. (2018). Staging and grading of periodontitis: Framework and proposal of a new classification and case definition. J. Periodontol..

[2] Petersen P.E., Ogawa H. (2012). The global burden of periodontal disease: Towards integration with chronic disease prevention and control. Periodontology 2000.

[3] Papapanou P.N. (1999). Epidemiology of periodontal diseases: An update. J. Int. Acad. Periodontol..

[4] Genco R.J., Sanz M. (2020). Clinical and public health implications of periodontal and systemic diseases: An overview. Periodontology 2000.

[5] Sanz M., Lang N.P., Kinane D.F., Berglundh T., Chapple I., Tonetti M.S. (2011). Seventh European Workshop on Periodontology of the European Academy of Periodontology at the Parador at la Granja, Segovia, Spain. J. Clin. Periodontol..

[6] Bui F.Q., Almeida-da-Silva C.L.C., Huynh B., Trinh A., Liu J., Woodward J., Asadi H., Ojcius D.M. (2019). Association between periodontal pathogens and systemic disease. Biomed. J..

[7] Sanz M., Kornman K. (2013). Periodontitis and adverse pregnancy outcomes: Consensus report of the Joint EFP/AAP Workshop on Periodontitis and Systemic Diseases. J. Periodontol..

[8] Matarese G., Isola G., Anastasi G.P., Cutroneo G., Cordasco G., Favaloro A., Vita G., Vermiglio G., Milardi D., Zizzari V.L. (2013). Transforming growth factor beta 1 and vascular endothelial growth factor levels in the pathogenesis of periodontal disease. Eur. J. Inflamm..

[9] Lertpimonchai A., Rattanasiri S., Arj-Ong Vallibhakara S., Attia J., Thakkinstian A. (2017). The association between oral hygiene and periodontitis: A systematic review and meta-analysis. Int. Dent. J..

[10] Cekici A., Kantarci A., Hasturk H., Van Dyke T.E. (2014). Inflammatory and immune pathways in the pathogenesis of periodontal disease. Periodontology 2000.

[11] Socransky S.S., Haffajee A.D. (2005). Periodontal microbial ecology. Periodontology 2000.

[12] Socransky S.S., Haffajee A.D., Cugini M.A., Smith C., Kent R.L. (1998). Microbial complexes in subgingival plaque. J. Clin. Periodontol..

[13] Bagavad Gita J., George A.V., Pavithra N., Chandrasekaran S.C., Latchumanadhas K., Gnanamani A. (2019). Dysregulation of miR-146a by periodontal pathogens: A risk for acute coronary syndrome. J. Periodontol..

[14] Preshaw P.M., Alba A.L., Herrera D., Jepsen S., Konstantinidis A., Makrilakis K., Taylor R. (2012). Periodontitis and diabetes: A two-way relationship. Diabetologia.

[15] Bianconi V., Sahebkar A., Atkin S.L., Pirro M. (2018). The regulation and importance of monocyte chemoattractant protein-1. Curr. Opin. Hematol..

[16] Yan K., Lin Q., Tang K., Liu S., Du Y., Yu X., Li S. (2020). Substance P participates in periodontitis by upregulating HIF-1alpha and RANKL/OPG ratio. BMC Oral Health.

[17] Ye D., Gajendra S., Lawyer G., Jadeja N., Pishey D., Pathagunti S., Lyons J., Veazie P., Watson G., McIntosh S. (2020). Inflammatory biomarkers and growth factors in saliva and gingival crevicular fluid of e-cigarette users, cigarette smokers, and dual smokers: A pilot study. J. Periodontol..

[18] Mittal M., Siddiqui M.R., Tran K., Reddy S.P., Malik A.B. (2014). Reactive oxygen species in inflammation and tissue injury. Antioxid. Redox Signal..

[19] Eick S., Nydegger J., Bürgin W., Salvi G.E., Sculean A., Ramseier C. (2018). Microbiological analysis and the outcomes of periodontal treatment with or without adjunctive systemic antibiotics—A retrospective study. Clin. Oral Investig..

[20] Franco C., Patricia H.R., Timo S., Claudia B., Marcela H. (2017). Matrix Metalloproteinases as Regulators of Periodontal Inflammation. Int. J. Mol. Sci..

[21] Becerra-Ruiz J.S., Guerrero-Velázquez C., Martínez-Esquivias F., Martínez-Pérez L.A., Guzmán-Flores J.M. (2021). Innate and adaptive immunity of periodontal disease. From etiology to alveolar bone loss. Oral Dis..

[22] Federle M.J., Bassler B.L. (2003). Interspecies communication in bacteria. J. Clin. Investig..

[23] Muras A., Mallo N., Otero-Casal P., Pose-Rodríguez J.M., Otero A. (2022). Quorum sensing systems as a new target to prevent biofilm-related oral diseases. Oral Dis..

[24] Souza E.Q.M., da Rocha T.E., Toro L.F., Guiati I.Z., Ervolino E., Garcia V.G., Wainwright M., Theodoro L.H. (2020). Antimicrobial photodynamic therapy compared to systemic antibiotic therapy in non-surgical treatment of periodontitis: Systematic review and meta-analysis. Photodiagnosis Photodyn. Ther..

[25] Isola G., Polizzi A., Iorio-Siciliano V., Alibrandi A., Ramaglia L., Leonardi R. (2021). Effectiveness of a nutraceutical agent in the non-surgical periodontal therapy: A randomized, controlled clinical trial. Clin. Oral Investig..

[26] Mummolo S., Marchetti E., Di Martino S., Scorzetti L., Marzo G. (2008). Aggressive periodontitis: Laser Nd:YAG treatment versus conventional surgical therapy. Eur. J. Paediatr. Dent..

[27] Khattri S., Nagraj S.K., Arora A., Eachempati P., Kusum C.K., Bhat K.G., Johnson T.M., Lodi G. (2020). Adjunctive systemic antimicrobials for the non-surgical treatment of periodontitis. Cochrane Database Syst. Rev..

[28] Morozumi T., Yashima A., Gomi K., Ujiie Y., Izumi Y., Akizuki T., Mizutani K., Takamatsu H., Minabe M., Miyauchi S. (2018). Increased systemic levels of inflammatory mediators following one-stage full-mouth scaling and root planing. J. Periodontal Res..

[29] Basavaraju M., Sisnity V.S., Palaparthy R., Addanki P.K. (2016). Quorum quenching: Signal jamming in dental plaque biofilms. J. Dent. Sci..

[30] Czaczyk K., Myszka K. (2007). Biosynthesis of extracellular polymeric substances (EPS) and its role in microbial biofilm formation. Pol. J. Environ. Stud..

[31] Hao Y., Winans S.C., Glick B.R., Charles T.C. (2010). Identification and characterization of new LuxR/LuxI-type quorum sensing systems from metagenomic libraries. Environ. Microbiol..

[32] Plančak D., Musić L., Puhar I. (2015). Quorum sensing of periodontal pathogens. Acta Stomatol. Croat..

[33] Papenfort K., Bassler B.L. (2016). Quorum sensing signal–response systems in Gram-negative bacteria. Nat. Rev. Microbiol..

[34] Reading N.C., Sperandio V. (2006). Quorum sensing: The many languages of bacteria. FEMS Microbiol. Lett..

[35] Nealson K.H., Hastings J.W. (1979). Bacterial bioluminescence: Its control and ecological significance. Microbiol. Rev..

[36] Bassler B.L., Wright M., Showalter R.E., Silverman M.R. (1993). Intercellular signalling in Vibrio harveyi: Sequence and function of genes regulating expression of luminescence. Mol. Microbiol..

[37] Bassler B.L., Losick R. (2006). Bacterially speaking. Cell.

[38] Petersen F.C., Pecharki D., Scheie A.A. (2004). Biofilm mode of growth of Streptococcus intermedius favored by a competence-stimulating signaling peptide. J. Bacteriol..

[39] Waters C.M., Bassler B.L. (2005). Quorum sensing: Cell-to-cell communication in bacteria. Annu. Rev. Cell Dev. Biol..

[40] (2010). Determinants of pulse wave velocity in healthy people and in the presence of cardiovascular risk factors: ‘Establishing normal and reference values’. Eur. Heart J..

[41] Li Y.H., Lau P.C., Lee J.H., Ellen R.P., Cvitkovitch D.G. (2001). Natural genetic transformation of *Streptococcus mutans* growing in biofilms. J. Bacteriol..

[42] Li Y.H., Tang N., Aspiras M.B., Lau P.C., Lee J.H., Ellen R.P., Cvitkovitch D.G. (2002). A quorum-sensing signaling system essential for genetic competence in Streptococcus mutans is involved in biofilm formation. J. Bacteriol..

[43] Ishii S., Fukui K., Yokoshima S., Kumagai K., Beniyama Y., Kodama T., Fukuyama T., Okabe T., Nagano T., Kojima H. (2017). High-throughput Screening of Small Molecule Inhibitors of the Streptococcus Quorum-sensing Signal Pathway. Sci. Rep..

[44] Li Y.H., Lau P.C., Tang N., Svensäter G., Ellen R.P., Cvitkovitch D.G. (2002). Novel two-component regulatory system involved in biofilm formation and acid resistance in *Streptococcus mutans*. J. Bacteriol..

[45] Xie Z., Okinaga T., Niu G., Qi F., Merritt J. (2010). Identification of a novel bacteriocin regulatory system in *Streptococcus mutans*. Mol. Microbiol..

[46] Shanker E., Federle M.J. (2017). Quorum Sensing Regulation of Competence and Bacteriocins in Streptococcus pneumoniae and mutans. Genes.

[47] Rued B.E., Covington B.C., Bushin L.B., Szewczyk G., Laczkovich I., Seyedsayamdost M.R., Federle M.J. (2021). Quorum Sensing in *Streptococcus mutans* Regulates Production of Tryglysin, a Novel RaS-RiPP Antimicrobial Compound. mBio.

[48] Bassler B.L. (1999). How bacteria talk to each other: Regulation of gene expression by quorum sensing. Curr. Opin. Microbiol..

[49] Wright P.P., Ramachandra S.S. (2022). Quorum Sensing and Quorum Quenching with a Focus on Cariogenic and Periodontopathic Oral Biofilms. Microorganisms.

[50] Frias J., Olle E., Alsina M. (2001). Periodontal pathogens produce quorum sensing signal molecules. Infect. Immun..

[51] Wang B.Y., Alvarez P., Hong J., Kuramitsu H.K. (2011). Periodontal pathogens interfere with quorum-sensing-dependent virulence properties in *Streptococcus mutans*. J. Periodontal. Res..

[52] Bachtiar E.W., Bachtiar B.M., Jarosz L.M., Amir L.R., Sunarto H., Ganin H., Meijler M.M., Krom B.P. (2014). AI-2 of Aggregatibacter actinomycetemcomitans inhibits *Candida albicans* biofilm formation. Front. Cell. Infect. Microbiol..

[53] Wang X., Li X., Ling J. (2017). Streptococcus gordonii LuxS/autoinducer-2 quorum-sensing system modulates the dual-species biofilm formation with *Streptococcus mutans*. J. Basic Microbiol..

[54] Koo H., Andes D.R., Krysan D.J. (2018). Candida-streptococcal interactions in biofilm-associated oral diseases. PLoS Pathog..

[55] Kumari A., Pasini P., Daunert S. (2008). Detection of bacterial quorum sensing N-acyl homoserine lactones in clinical samples. Anal. Bioanal. Chem..

[56] Kumari A., Pasini P., Deo S.K., Flomenhoft D., Shashidhar H., Daunert S. (2006). Biosensing systems for the detection of bacterial quorum signaling molecules. Anal. Chem..

[57] Muras A., Mayer C., Otero-Casal P., Exterkate R.A.M., Brandt B.W., Crielaard W., Otero A., Krom B.P. (2020). Short-Chain N-Acylhomoserine Lactone Quorum-Sensing Molecules Promote Periodontal Pathogens in In Vitro Oral Biofilms. Appl. Environ. Microbiol..

[58] Hajishengallis G., Darveau R.P., Curtis M.A. (2012). The keystone-pathogen hypothesis. Nat. Rev. Microbiol..

[59] Kolenbrander P.E., Palmer R.J., Rickard A.H., Jakubovics N.S., Chalmers N.I., Diaz P.I. (2006). Bacterial interactions and successions during plaque development. Periodontology 2000.

[60] Kolenbrander P.E., Andersen R.N., Blehert D.S., Egland P.G., Foster J.S., Palmer R.J. (2002). Communication among oral bacteria. Microbiol. Mol. Biol. Rev..

[61] Rickard A.H., Palmer R.J., Blehert D.S., Campagna S.R., Semmelhack M.F., Egland P.G., Bassler B.L., Kolenbrander P.E. (2006). Autoinducer 2: A concentration-dependent signal for mutualistic bacterial biofilm growth. Mol. Microbiol..

[62] Shao H., Lamont R.J., Demuth D.R. (2007). Autoinducer 2 is required for biofilm growth of *Aggregatibacter* (*Actinobacillus*) *actinomycetemcomitans*. Infect. Immun..

[63] James D., Shao H., Lamont R.J., Demuth D.R. (2006). The Actinobacillus actinomycetemcomitans ribose binding protein RbsB interacts with cognate and heterologous autoinducer 2 signals. Infect. Immun..

[64] Shao H., James D., Lamont R.J., Demuth D.R. (2007). Differential interaction of *Aggregatibacter* (*Actinobacillus*) *actinomycetemcomitans* LsrB and RbsB proteins with autoinducer 2. J. Bacteriol..

[65] Fine D.H., Velliyagounder K., Furgang D., Kaplan J.B. (2005). The Actinobacillus actinomycetemcomitans autotransporter adhesin Aae exhibits specificity for buccal epithelial cells from humans and old world primates. Infect. Immun..

[66] Rupani D., Izano E.A., Schreiner H.C., Fine D.H., Kaplan J.B. (2008). Aggregatibacter actinomycetemcomitans serotype f O-polysaccharide mediates coaggregation with Fusobacterium nucleatum. Oral Microbiol. Immunol..

[67] Szafrański S.P., Deng Z.L., Tomasch J., Jarek M., Bhuju S., Rohde M., Sztajer H., Wagner-Döbler I. (2017). Quorum sensing of Streptococcus mutans is activated by Aggregatibacter actinomycetemcomitans and by the periodontal microbiome. BMC Genom..

[68] Fong K.P., Gao L., Demuth D.R. (2003). luxS and arcB control aerobic growth of Actinobacillus actinomycetemcomitans under iron limitation. Infect. Immun..

[69] James C.E., Hasegawa Y., Park Y., Yeung V., Tribble G.D., Kuboniwa M., Demuth D.R., Lamont R.J. (2006). LuxS involvement in the regulation of genes coding for hemin and iron acquisition systems in Porphyromonas gingivalis. Infect. Immun..

[70] McNab R., Ford S.K., El-Sabaeny A., Barbieri B., Cook G.S., Lamont R.J. (2003). LuxS-based signaling in Streptococcus gordonii: Autoinducer 2 controls carbohydrate metabolism and biofilm formation with Porphyromonas gingivalis. J. Bacteriol..

[71] Yuan L., Hillman J.D., Progulske-Fox A. (2005). Microarray analysis of quorum-sensing-regulated genes in Porphyromonas gingivalis. Infect. Immun..

[72] Jang Y.J., Choi Y.J., Lee S.H., Jun H.K., Choi B.K. (2013). Autoinducer 2 of Fusobacterium nucleatum as a target molecule to inhibit biofilm formation of periodontopathogens. Arch. Oral Biol..

[73] Jang Y.J., Sim J., Jun H.K., Choi B.K. (2013). Differential effect of autoinducer 2 of Fusobacterium nucleatum on oral streptococci. Arch. Oral Biol..

[74] Azakami H., Teramura I., Matsunaga T., Akimichi H., Noiri Y., Ebisu S., Kato A. (2006). Characterization of autoinducer 2 signal in Eikenella corrodens and its role in biofilm formation. J. Biosci. Bioeng..

[75] Muras A., Otero-Casal P., Blanc V., Otero A. (2020). Acyl homoserine lactone-mediated quorum sensing in the oral cavity: A paradigm revisited. Sci. Rep..

[76] Baehni P.C., Takeuchi Y. (2003). Anti-plaque agents in the prevention of biofilm-associated oral diseases. Oral Dis..

[77] (2002). Mouthrinses and periodontal disease. Int. Dent. J..

[78] Herrera D., Sanz M., Jepsen S., Needleman I., Roldán S. (2002). A systematic review on the effect of systemic antimicrobials as an adjunct to scaling and root planing in periodontitis patients. J. Clin. Periodontol..

[79] Ehmke B., Moter A., Beikler T., Milian E., Flemmig T.F. (2005). Adjunctive antimicrobial therapy of periodontitis: Long-term effects on disease progression and oral colonization. J. Periodontol..

[80] Mombelli A., Cionca N., Almaghlouth A. (2011). Does adjunctive antimicrobial therapy reduce the perceived need for periodontal surgery?. Periodontology 2000.

[81] Griffiths G.S., Ayob R., Guerrero A., Nibali L., Suvan J., Moles D.R., Tonetti M.S. (2011). Amoxicillin and metronidazole as an adjunctive treatment in generalized aggressive periodontitis at initial therapy or re-treatment: A randomized controlled clinical trial. J. Clin. Periodontol..

[82] Kaner D., Christan C., Dietrich T., Bernimoulin J.P., Kleber B.M., Friedmann A. (2007). Timing affects the clinical outcome of adjunctive systemic antibiotic therapy for generalized aggressive periodontitis. J. Periodontol..

[83] Ribeiro Edel P., Bittencourt S., Zanin I.C., Bovi Ambrosano G.M., Sallum E.A., Nociti F.H., Gonçalves R.B., Casati M.Z. (2009). Full-mouth ultrasonic debridement associated with amoxicillin and metronidazole in the treatment of severe chronic periodontitis. J. Periodontol..

[84] Kalsi R., Vandana K.L., Prakash S. (2011). Effect of local drug delivery in chronic periodontitis patients: A meta-analysis. J. Indian Soc. Periodontol..

[85] Lang N.P., Tan W.C., Krähenmann M.A., Zwahlen M. (2008). A systematic review of the effects of full-mouth debridement with and without antiseptics in patients with chronic periodontitis. J. Clin. Periodontol..

[86] Berglundh T., Giannobile W.V., Sanz M., Lang N.P. (2021). Lindhe’s Clinical Periodontology and Implant Dentistry.

[87] James P., Worthington H.V., Parnell C., Harding M., Lamont T., Cheung A., Whelton H., Riley P. (2017). Chlorhexidine mouthrinse as an adjunctive treatment for gingival health. Cochrane Database Syst. Rev..

[88] Zhao H., Hu J., Zhao L. (2020). Adjunctive subgingival application of Chlorhexidine gel in nonsurgical periodontal treatment for chronic periodontitis: A systematic review and meta-analysis. BMC Oral Health.

[89] Brookes Z.L.S., Bescos R., Belfield L.A., Ali K., Roberts A. (2020). Current uses of chlorhexidine for management of oral disease: A narrative review. J. Dent..

[90] Stoeken J.E., Paraskevas S., van der Weijden G.A. (2007). The long-term effect of a mouthrinse containing essential oils on dental plaque and gingivitis: A systematic review. J. Periodontol..

[91] Ciancio S.G. (2000). Antiseptics and antibiotics as chemotherapeutic agents for periodontitis management. Compend. Contin. Educ. Dent..

[92] Gunsolley J.C. (2006). A meta-analysis of six-month studies of antiplaque and antigingivitis agents. J. Am. Dent. Assoc..

[93] Chee B., Park B., Fitzsimmons T., Coates A.M., Bartold P.M. (2016). Omega-3 fatty acids as an adjunct for periodontal therapy-a review. Clin. Oral Investig..

[94] Kruse A.B., Kowalski C.D., Leuthold S., Vach K., Ratka-Krüger P., Woelber J.P. (2020). What is the impact of the adjunctive use of omega-3 fatty acids in the treatment of periodontitis? A systematic review and meta-analysis. Lipids Health Dis..

[95] Heo H., Bae J.H., Amano A., Park T., Choi Y.H. (2022). Supplemental or dietary intake of omega-3 fatty acids for the treatment of periodontitis: A meta-analysis. J. Clin. Periodontol..

[96] Mayanagi G., Kimura M., Nakaya S., Hirata H., Sakamoto M., Benno Y., Shimauchi H. (2009). Probiotic effects of orally administered Lactobacillus salivarius WB21-containing tablets on periodontopathic bacteria: A double-blinded, placebo-controlled, randomized clinical trial. J. Clin. Periodontol..

[97] Nguyen T., Brody H., Radaic A., Kapila Y. (2021). Probiotics for periodontal health—Current molecular findings. Periodontology 2000.

[98] Rehman Z.U., Leiknes T. (2018). Quorum-Quenching Bacteria Isolated From Red Sea Sediments Reduce Biofilm Formation by Pseudomonas aeruginosa. Front. Microbiol..

[99] Murugayah S.A., Gerth M.L. (2019). Engineering quorum quenching enzymes: Progress and perspectives. Biochem. Soc. Trans..

[100] Lade H., Paul D., Kweon J.H. (2014). Quorum quenching mediated approaches for control of membrane biofouling. Int. J. Biol. Sci..

[101] Sikdar R., Elias M. (2020). Quorum quenching enzymes and their effects on virulence, biofilm, and microbiomes: A review of recent advances. Expert Rev. Anti. Infect. Ther..

[102] Ni N., Li M., Wang J., Wang B. (2009). Inhibitors and antagonists of bacterial quorum sensing. Med. Res. Rev..

[103] Rampioni G., Leoni L., Williams P. (2014). The art of antibacterial warfare: Deception through interference with quorum sensing-mediated communication. Bioorg. Chem..

[104] Watson W.T., Minogue T.D., Val D.L., von Bodman S.B., Churchill M.E. (2002). Structural basis and specificity of acyl-homoserine lactone signal production in bacterial quorum sensing. Mol. Cell.

[105] Rutherford S.T., Bassler B.L. (2012). Bacterial quorum sensing: Its role in virulence and possibilities for its control. Cold Spring Harb. Perspect. Med..

[106] Asahi Y., Noiri Y., Igarashi J., Asai H., Suga H., Ebisu S. (2010). Effects of N-acyl homoserine lactone analogues on *Porphyromonas gingivalis* biofilm formation. J. Periodontal Res..

[107] Uroz S., Oger P.M., Chapelle E., Adeline M.T., Faure D., Dessaux Y. (2008). A Rhodococcus qsdA-encoded enzyme defines a novel class of large-spectrum quorum-quenching lactonases. Appl. Environ. Microbiol..

[108] Leadbetter J.R., Greenberg E.P. (2000). Metabolism of acyl-homoserine lactone quorum-sensing signals by Variovorax paradoxus. J. Bacteriol..

[109] Koch G., Nadal-Jimenez P., Reis C.R., Muntendam R., Bokhove M., Melillo E., Dijkstra B.W., Cool R.H., Quax W.J. (2014). Reducing virulence of the human pathogen Burkholderia by altering the substrate specificity of the quorum-quenching acylase PvdQ. Proc. Natl. Acad. Sci. USA.

[110] Bijtenhoorn P., Mayerhofer H., Müller-Dieckmann J., Utpatel C., Schipper C., Hornung C., Szesny M., Grond S., Thürmer A., Brzuszkiewicz E.B. (2011). A Novel Metagenomic Short-Chain Dehydrogenase/Reductase Attenuates *Pseudomonas aeruginosa* Biofilm Formation and Virulence on Caenorhabditis elegans. PLoS ONE.

[111] Dwivedi D., Singh V. (2016). Effects of the natural compounds embelin and piperine on the biofilm-producing property of Streptococcus mutans. J. Tradit. Complement. Med..

[112] Fournier-Larente J., Morin M.P., Grenier D. (2016). Green tea catechins potentiate the effect of antibiotics and modulate adherence and gene expression in Porphyromonas gingivalis. Arch. Oral. Biol..

[113] Yada S., Kamalesh B., Sonwane S., Guptha I., Swetha R.K. (2015). Quorum sensing inhibition, relevance to periodontics. J. Int. Oral Health.

[114] Amin A., Hanif M., Abbas K., Ramzan M., Rasheed A., Zaman A., Pieters L. (2020). Studies on effects of umbelliferon derivatives against periodontal bacteria; antibiofilm, inhibition of quorum sensing and molecular docking analysis. Microb. Pathog..

[115] Asfour H.Z. (2018). Anti-Quorum Sensing Natural Compounds. J. Microsc. Ultrastruct..

[116] Dong Y.H., Wang L.Y., Zhang L.H. (2007). Quorum-quenching microbial infections: Mechanisms and implications. Philos. Trans. R Soc. Lond. B Biol. Sci..

[117] González J.E., Keshavan N.D. (2006). Messing with Bacterial Quorum Sensing. Microbiol. Mol. Biol. Rev..

[118] Park J.S., Ryu E.J., Li L., Choi B.K., Kim B.M. (2017). New bicyclic brominated furanones as potent autoinducer-2 quorum-sensing inhibitors against bacterial biofilm formation. Eur. J. Med. Chem..

[119] Ponnusamy K., Paul D., Sam Kim Y., Kweon J.H. (2010). 2(5H)-Furanone: A Prospective strategy for biofouling-control in membrane biofilm bacteria by quorum sensing inhibition. Braz. J. Microbiol..

[120] Cho Y.J., Song H.Y., Ben Amara H., Choi B.K., Eunju R., Cho Y.A., Seol Y., Lee Y., Ku Y., Rhyu I.C. (2016). In Vivo Inhibition of *Porphyromonas gingivalis* Growth and Prevention of Periodontitis With Quorum-Sensing Inhibitors. J. Periodontol..

[121] Ryu E.J., Sim J., Sim J., Lee J., Choi B.K. (2016). D-Galactose as an autoinducer 2 inhibitor to control the biofilm formation of periodontopathogens. J. Microbiol..

[122] Guendouze A., Plener L., Bzdrenga J., Jacquet P., Rémy B., Elias M., Lavigne J.-P., Daudé D., Chabrière E. (2017). Effect of Quorum Quenching Lactonase in Clinical Isolates of *Pseudomonas aeruginosa* and Comparison with Quorum Sensing Inhibitors. Front. Microbiol..

[123] Ivanova K., Fernandes M.M., Mendoza E., Tzanov T. (2015). Enzyme multilayer coatings inhibit *Pseudomonas aeruginosa* biofilm formation on urinary catheters. Appl. Microbiol. Biotechnol..

[124] Schwab M., Bergonzi C., Sakkos J., Staley C., Zhang Q., Sadowsky M.J., Aksan A., Elias M. (2019). Signal Disruption Leads to Changes in Bacterial Community Population. Front. Microbiol..

[125] Simonetti O., Cirioni O., Cacciatore I., Baldassarre L., Orlando F., Pierpaoli E., Lucarini G., Orsetti E., Provinciali M., Fornasari E. (2016). Efficacy of the Quorum Sensing Inhibitor FS10 Alone and in Combination with Tigecycline in an Animal Model of Staphylococcal Infected Wound. PLoS ONE.

[126] Paluch E., Rewak-Soroczyńska J., Jędrusik I., Mazurkiewicz E., Jermakow K. (2020). Prevention of biofilm formation by quorum quenching. Appl. Microbiol. Biotechnol..

[127] Abranches J., Zeng L., Kajfasz J.K., Palmer S.R., Chakraborty B., Wen Z.T., Richards V.P., Brady L.J., Lemos J.A. (2018). Biology of Oral Streptococci. Microbiol. Spectr..

[128] Park O.J., Kwon Y., Park C., So Y.J., Park T.H., Jeong S., Im J., Yun C.H., Han S.H. (2020). *Streptococcus gordonii*: Pathogenesis and Host Response to Its Cell Wall Components. Microorganisms.

[129] Loo C.Y., Corliss D.A., Ganeshkumar N. (2000). *Streptococcus gordonii* biofilm formation: Identification of genes that code for biofilm phenotypes. J. Bacteriol..

[130] Park T., Im J., Kim A.R., Lee D., Jeong S., Yun C.H., Han S.H. (2021). Short-chain fatty acids inhibit the biofilm formation of *Streptococcus gordonii* through negative regulation of competence-stimulating peptide signaling pathway. J. Microbiol..

[131] OmerOglou E., Karaca B., Kibar H., Haliscelik O., Kiran F. (2022). The role of microbiota-derived postbiotic mediators on biofilm formation and quorum sensing-mediated virulence of *Streptococcus mutans*: A perspective on preventing dental caries. Microb. Pathog..

[132] Kaspar J.R., Walker A.R. (2019). Expanding the Vocabulary of Peptide Signals in *Streptococcus mutans*. Front. Cell. Infect. Microbiol..

[133] Vijayakumar A., Sarveswari H.B., Vasudevan S., Shanmugam K., Solomon A.P., Neelakantan P. (2021). Baicalein Inhibits *Streptococcus mutans* Biofilms and Dental Caries-Related Virulence Phenotypes. Antibiotics.

[134] Balasubramanian A.R., Vasudevan S., Shanmugam K., Lévesque C.M., Solomon A.P., Neelakantan P. (2021). Combinatorial effects of trans-cinnamaldehyde with fluoride and chlorhexidine on *Streptococcus mutans*. J. Appl. Microbiol..

[135] Ogawa A., Furukawa S., Fujita S., Mitobe J., Kawarai T., Narisawa N., Sekizuka T., Kuroda M., Ochiai K., Ogihara H. (2011). Inhibition of Streptococcus mutans biofilm formation by *Streptococcus salivarius* FruA. Appl. Environ. Microbiol..

[136] Wasfi R., Abd El-Rahman O.A., Zafer M.M., Ashour H.M. (2018). Probiotic *Lactobacillus* sp. inhibit growth, biofilm formation and gene expression of caries-inducing Streptococcus mutans. J. Cell. Mol. Med..

[137] Kasper S.H., Samarian D., Jadhav A.P., Rickard A.H., Musah R.A., Cady N.C. (2014). S-aryl-L-cysteine sulphoxides and related organosulphur compounds alter oral biofilm development and AI-2-based cell-cell communication. J. Appl. Microbiol..

[138] Elani H.W., Starr J.R., Da Silva J.D., Gallucci G.O. (2018). Trends in Dental Implant Use in the U.S., 1999-2016, and Projections to 2026. J. Dent. Res..

[139] Ramachandra S.S., Rana R., Reetika S., Jithendra K.D. (2014). Options to avoid the second surgical site: A review of literature. Cell Tissue Bank.

[140] Heitz-Mayfield L.J.A., Salvi G.E. (2018). Peri-implant mucositis. J. Clin. Periodontol..

[141] Renvert S., Persson G.R., Pirih F.Q., Camargo P.M. (2018). Peri-implant health, peri-implant mucositis, and peri-implantitis: Case definitions and diagnostic considerations. J. Periodontol..

[142] Kang M., Kim S., Kim H., Song Y., Jung D., Kang S., Seo J.H., Nam S., Lee Y. (2019). Calcium-Binding Polymer-Coated Poly(lactide- co-glycolide) Microparticles for Sustained Release of Quorum Sensing Inhibitors to Prevent Biofilm Formation on Hydroxyapatite Surfaces. ACS Appl. Mater. Interfaces.

[143] An S.-J., Namkung J.-U., Ha K.-W., Jun H.-K., Kim H.Y., Choi B.-K. (2022). Inhibitory effect of d-arabinose on oral bacteria biofilm formation on titanium discs. Anaerobe.

[144] Hemmati F., Rezaee M.A., Ebrahimzadeh S., Yousefi L., Nouri R., Kafil H.S., Gholizadeh P. (2021). Novel Strategies to Combat Bacterial Biofilms. Mol. Biotechnol..

[145] Kim W., Soh Y., Heo S.-M. (2021). Recent Advances of Therapeutic Targets for the Treatment of Periodontal Disease. Biomol. Ther..

